# EEG Microstate Alterations in Eyes-Open and Eyes-Closed Resting States Across the Alzheimer’s Disease Continuum

**DOI:** 10.3390/jcm15155863

**Published:** 2026-07-27

**Authors:** Chanda Simfukwe, Seong Soo A. An, Young Chul Youn

**Affiliations:** 1 Department of Bionano Technology, Gachon University, Seongnam-si 1342, Republic of Korea; chandaelizabeth94@gmail.com; 2 Department of Neurology, College of Medicine, Chung-Ang University, Seoul 06974, Republic of Korea; 3 Department of Medical Informatics, Chung-Ang University College of Medicine, Seoul 06974, Republic of Korea

**Keywords:** Alzheimer’s disease, cognitive dysfunction, electroencephalography, microstates, aging

## Abstract

**Background/Objective**: Quantitative electroencephalography (qEEG) microstates, recorded under both eyes-open (EOR) and eyes-closed (ECR) resting conditions, provide a powerful neurophysiological approach for capturing the temporal dynamics of cognitive processing. Nevertheless, microstate syntax remains poorly delineated in mild cognitive impairment (MCI) and Alzheimer’s disease (AD). The present study seeks to identify potential drivers of altered microstate topography and temporal dynamics across advancing stages of cognitive decline. **Methods**: Resting-state EEG (rEEG) was recorded from 60 participants (40–90 years old) in each of three groups: MCI, AD, and healthy controls (HC), under both EOR and ECR conditions. After artifact rejection with EEGLAB in MATLAB R2024a, the final dataset comprised 180 clean recordings per condition (EOR and ECR) and 360 recordings in total. Microstate analysis was performed using the MICROSTATELAB toolbox. Potential group differences in microstate topography were examined with topographic analysis of variance (TANOVA). Four canonical microstates were extracted and labeled A, B, C, and E, following the well-established classification scheme. **Results**: Analysis of microstate topographies revealed significant group-level differences in the EOR condition for Microstate A (auditory network; *p* = 0.003), Microstate C (salience network; *p* = 0.004), and Microstate E (executive network; *p* = 0.036). In contrast, no significant between-group differences emerged under the ECR condition (all *p* > 0.05). Within-group comparisons indicated that Microstate B (visual network) was the only class to differ between the two resting conditions in healthy controls and patients with MCI. In the AD group, however, condition-related differences extended to Microstates A, C, and E, suggesting a more distributed disruption of network dynamics in advanced disease. Temporally, Microstate B coverage was the only parameter to show a significant between-group difference, being greater in MCI than in AD under the EOR condition (*p* = 0.031); visual trends of increasing Microstate A duration and decreasing Microstate C occurrence toward AD did not reach statistical significance. **Conclusions**: This study’s rEEG microstate analysis uncovered condition-dependent and disease-sensitive alterations across the HC, MCI, and AD groups. The EOR condition proved more diagnostically informative, with significant group-level topographic differences emerging for Microstates A, C, and E, whereas the ECR condition yielded no reliable between-group effects. Microstate B coverage showed the only significant temporal alteration, distinguishing MCI from AD under EOR, while Microstate A and Microstate C showed non-significant trends warranting replication in larger cohorts.

## 1. Introduction

Alzheimer’s disease (AD) is a progressive neurodegenerative condition marked by multi-domain cognitive deterioration spanning memory, executive function, language, and visuospatial abilities [1,2]. As the leading cause of dementia, AD accounts for approximately 70% of cases worldwide and imposes a growing burden on patients, families, and healthcare systems [3,4]. Many individuals first transition through a prodromal phase termed mild cognitive impairment (MCI), in which cognitive decline exceeds that expected with normal aging but remains below the clinical threshold for dementia [5,6]. Despite extensive research, the neurophysiological processes linking stage-specific cognitive deterioration to altered brain network activity remain incompletely understood.

Electroencephalography (EEG) non-invasively captures cortical dynamics at the millisecond timescale, a resolution well suited to the neural computations that underlie cognition [7]. Applying quantitative signal-processing methods to standard EEG recordings, an approach known as quantitative EEG (qEEG), enables neurophysiological biomarkers to be extracted with greater objectivity and precision [8,9]. Among qEEG techniques, resting-state EEG (rEEG) microstate analysis has attracted particular interest for characterizing the temporal organization of large-scale brain networks in relation to cognitive function [10,11,12]. rEEG microstates are commonly defined as quasi-stable scalp potential topographies, typically derived from alpha-band activity (8–12 Hz), each persisting for approximately 60–120 ms before transitioning to another state [13,14]. The widely adopted four-class solution delineates Classes A through D, corresponding, respectively, to auditory-phonological, visual, salience, and frontoparietal executive control networks [12,13,14], whereas extended solutions identify up to seven classes, with Classes E, F, and G capturing supplementary configurations [15]. Notably, Classes D and E exhibit substantial functional overlap and are frequently used interchangeably, with label assignment contingent on the clustering approach and the number of extracted classes [15,16].

These repeated states of brain electrical activity are thought to capture momentary states of spontaneous mental processing. In this sense, rEEG microstates have been described as “atoms of thought,” as they may represent short-lived but meaningful episodes in the organization of neural information processing [17]. Consistent with this interpretation, a convergent body of evidence has demonstrated robust associations between microstate parameters and a variety of cognitive and perceptual operations [18,19], underscoring the theoretical and translational significance of microstate analysis for the study of neurological disorders. The temporal organization of microstate dynamics is commonly characterized through three principal parameters: duration, occurrence, and coverage [20,21]. Duration refers to the mean time a microstate configuration remains stable before transitioning to a subsequent state and has been proposed to reflect the efficiency and flexibility of large-scale neural network engagement during spontaneous brain activity [22]. Occurrence denotes the rate at which a given microstate is reinstated per unit time and is understood to index the brain’s propensity to recruit specific resting-state networks [22,23]. Coverage represents the proportion of total recording time occupied by each microstate class and, by integrating both duration and occurrence, provides a composite measure of each network state’s overall dominance within the resting-state EEG signal [23,24].

Microstate duration is widely regarded as the most diagnostically sensitive temporal parameter, reflecting the stability and efficiency with which transient brain networks configure themselves. However, its behavior across the cognitive decline spectrum remains contested: early work found no differences in duration between AD and healthy controls (HC), whereas later studies found longer microstate duration associated with advancing disease severity in both MCI and AD [25,26,27,28,29]. Moving beyond isolated parameters, the sequential structure of microstates, or microstate syntax, offers a richer, systems-level lens on the temporal organization of spontaneous brain activity. This framework captures relational properties invisible to single-state metrics, revealing how microstates assemble into syllable-like transitions, word-like recurring sequences, and higher-order grammar-like regularities [28,29,30,31]. In the MCI and AD literature, syntactic analyses have been almost exclusively confined to pairwise transition probabilities, yielding a fragmented picture.

Nishida et al. (2013) [26] used EEG microstate transition analysis across frontotemporal dementia, schizophrenia, mild AD, and HC, showing diagnosis-specific alterations in preferred microstate sequences rather than a clearly random transition pattern in AD. In contrast, Musaeus et al. (2019) [22] applied a non-parametric randomization framework and found that transition probabilities in MCI and AD did not systematically deviate from randomness, despite robust changes in microstate occurrence and coverage. Later work by Schumacher et al. (2019) [32] identified non-random microstate transitions in Lewy body dementia, with AD patients showing microstate dynamics largely comparable to HC; the authors noted that cholinesterase inhibitor treatment could influence resting-state EEG patterns and potentially contribute to discrepancies between studies. Subsequent investigations have remained mixed: Musaeus et al. (2020) [33] reported disease-related alterations in specific microstate classes in MCI and AD, while other studies relying primarily on transition-probability metrics observed only modest between-group differences. Differences in medication status, disease phenotype, and analytical choices may therefore partly account for these divergent outcomes, though the precise microstate-level mechanisms remain poorly understood.

A further, largely overlooked limitation of the existing literature is the absence of systematic consideration of the resting-state recording condition. Prior studies have predominantly relied on a single resting paradigm without distinguishing between EOR and ECR states, two conditions known to produce fundamentally different patterns of neural activity and EEG topography [9,10]. To our knowledge, no study has yet examined how resting-state conditions modulate microstate topography and temporal dynamics in MCI and AD. This represents a substantive methodological gap, as condition-specific differences in microstate dynamics may partly explain inconsistencies across studies and carry important implications for the standardization of EEG microstate protocols in clinical research.

This study provides the first systematic comparison of resting-state microstate topography and temporal dynamics under both EOR and ECR conditions across the complete HC–MCI–AD continuum within a single, balanced cohort. This dual-condition design directly addresses a persistent methodological gap, as prior microstate studies in cognitive decline have relied almost exclusively on a single resting paradigm, thereby overlooking how the recording condition itself shapes disease-related network alterations. The present study aims to investigate the neurophysiological underpinnings of cognitive decline in MCI and AD using resting-state qEEG microstate analysis acquired under both EOR and ECR conditions. We hypothesize that: (1) microstate topography will differ significantly across diagnostic groups, with greater sensitivity observed under the EOR condition; (2) specific microstate classes will show systematic, stage-dependent alterations in temporal parameters that reflect progressive neural network dysfunction; and (3) distinct microstate configurations will differentially index disease progression versus modulation by resting-state condition.

## 2. Materials and Methods

### 2.1. Study Design

This study employed a cross-sectional, retrospective design based on data from a hospital-based cohort investigation that evaluated the prevalence of cognitive impairment and associated risk factors in an elderly population. Participants were stratified into three diagnostic groups, HC, MCI, and AD, and resting-state EEG microstate topography and temporal dynamics were compared across groups under both EOR and ECR conditions. An equal number of participants was included in each group to minimize group-size-related analytical bias (total N = 180).

### 2.2. Ethical Consent

All procedures were conducted in accordance with the ethical principles outlined in the Declaration of Helsinki and were approved by the Institutional Review Board of Chung-Ang University Hospital (IRB No. C2012049). As the study was retrospective and all data were blinded to personal information, the requirement for informed consent was waived by the Institutional Review Board of Chung-Ang University Hospital.

### 2.3. Study Population

**Figure 1:**
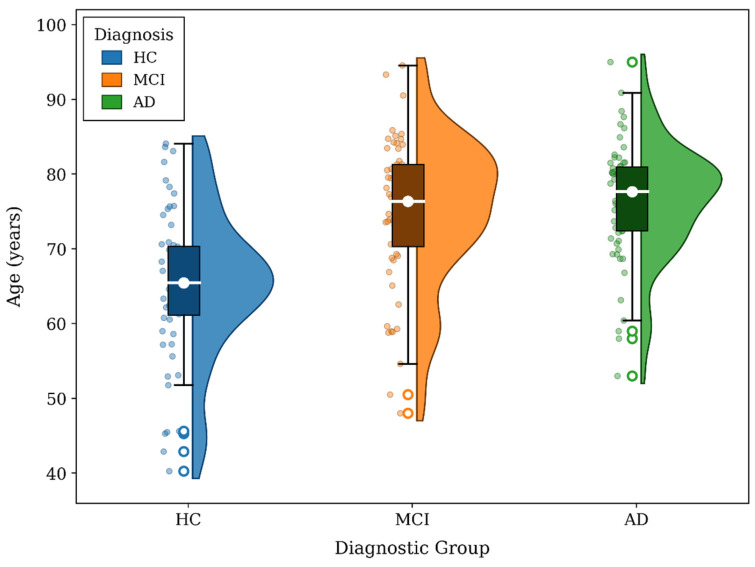
Distribution of participant age across the three diagnostic groups (HC, MCI, and AD; n = 60 per group). Data are displayed as raincloud plots, each combining three elements: a half-violin showing the kernel density estimate of the age distribution (right), a box plot (centre), and jittered individual participant data points (left). Boxes span the interquartile range, the white marker denotes the group median, and whiskers extend to 1.5× the interquartile range; open circles indicate values falling beyond the whisker range. Colours denote diagnostic group and are applied consistently throughout. The figure was generated in Python version 3.13.0 (28 March 2026). Abbreviations: HC, healthy controls; MCI, mild cognitive impairment; AD, Alzheimer’s disease.

A total of 180 participants were enrolled in the study, comprising three diagnostic groups: HC (n = 60), MCI (n = 60), and AD (n = 60), with ages ranging from 40 to 90 years. The mean ages of the HC, MCI, and AD groups were 64.54 ± 9.03, 73.95 ± 7.77, and 76.94 ± 8.03 years, respectively (mean ± standard deviation) (Figure 1).

Participants were excluded if magnetic resonance imaging (MRI) findings revealed neurological conditions, including cerebrovascular disease, epilepsy, psychosis, or focal structural brain lesions, deemed sufficient to explain the observed cognitive deficits independently. Additionally, individuals with a documented history of substance use disorder within the ten years preceding enrollment were ineligible for participation.

Inclusion criteria for the HC group were based on the 28 normative screening criteria for cognitively unimpaired older adults proposed by Christensen et al. [34], supplemented by quantitative neuropsychological thresholds: a Korean Mini-Mental State Examination (K-MMSE) score within or above −1 standard deviation (SD) of age-referenced norms, a Korean Instrumental Activities of Daily Living (K-IADL) score of ≤0.42, and a Korean Dementia Screening Questionnaire (KDSQ) score of ≤6 [35,36,37]. MCI classification required the presence of subjective memory complaints alongside objective cognitive performance falling within −1 SD of age- and education-stratified normative means across all domains assessed by the Seoul Neuropsychological Screening Battery (SNSB), in the absence of a dementia diagnosis according to the Diagnostic and Statistical Manual of Mental Disorders, Fourth Edition (DSM-IV) [38,39,40]. The SNSB is a standardized, comprehensive neuropsychological assessment developed and validated for Korean-speaking populations, encompassing multiple cognitive domains including attention, memory, language, executive function, and visuospatial processing. Patients with AD were diagnosed based on clinically documented progressive memory decline, functional impairment in activities of daily living, and behavioral or personality changes, supported by objective evidence of verbal memory deficits on the SNSB. Structural MRI was additionally performed to exclude intracranial pathology that could independently account for the observed cognitive deficits, thereby ensuring that impairment was attributable to AD rather than to confounding neuropathological conditions. All final diagnoses were established by a qualified neurologist following systematic neuropsychological evaluation and in accordance with recognized diagnostic criteria.

### 2.4. Resting-State EEG Acquisition and Preprocessing

The rEEG data were acquired using a Comet AS40 amplifier system (GRASS; Telefactor, West Warwick, RI, USA) with gold-cup electrodes positioned according to the international 10–20 system. A total of 19 electrodes were placed at standard scalp locations: Fp1, Fp2, F7, F3, Fz, F4, F8, T3, C3, Cz, C4, T4, T5, P3, Pz, P4, T6, O1, and O2, with bilateral earlobes serving as the reference (Supplementary Figure S1). Electrode-to-skin impedance was maintained below 5 kΩ throughout the recording to ensure signal integrity. All recordings were conducted in a quiet, controlled environment, with participants seated comfortably and instructed to remain relaxed and minimize voluntary movements, including blinking and swallowing, to reduce motion-related artifacts.

The EEG signals were digitally recorded and stored for offline analysis. Prior to data acquisition, an online bandpass filter from 0.5 to 100 Hz was applied to retain physiologically relevant neural activity while attenuating noise and out-of-band interference. Participants completed alternating blocks of EOR and ECR, with each condition comprising 10 trials, each lasting 30 s, yielding approximately 5 min of resting-state EEG per condition. Data were sampled at 250 Hz. Following preprocessing and artifact rejection, a minimum of 60 s of artifact-free data per condition was retained for each participant to ensure adequate spectral reliability. On average, 45 clean 4 s epochs, approximately 180 s of usable data, were included per participant and condition.

All resting-state EEG datasets were preprocessed using the EEGLAB toolbox (version 2024; http://www.sccn.ucsd.edu/eeglab/ (accessed on 14 March 2026)) implemented in MATLAB (R2024a). The preprocessing pipeline proceeded as follows. The EOR and ECR epochs were first extracted from the continuous raw signal. Common average re-referencing (CAR) was subsequently applied to minimize channel-level baseline variance across the electrode montage [41]. A bandpass filter with a passband of 1–4.5 Hz was applied to isolate the frequency range of interest while attenuating out-of-band noise. Bad epochs were rejected using Artifact Subspace Reconstruction (ASR), followed by automated artifact detection and baseline removal. Independent component analysis (ICA) using the Runica algorithm was then applied to identify and eliminate residual non-neural components, including ocular and muscular artifacts. Following the complete preprocessing pipeline, all 360 datasets were retained for microstate analysis, comprising 180 EOR and 180 ECR recordings across the three diagnostic groups (HC, MCI, and AD; n = 60 per group per condition).

### 2.5. EEG Microstate Analysis

Microstate analysis was performed using MICROSTATELAB version 2.1 (14 March 2026) [42], a validated open-source EEGLAB toolbox for standardized resting-state microstate analysis, implemented in MATLAB (R2024a), following previously established protocols [9]. Global field power (GFP), representing the instantaneous strength of the scalp electric field, was computed for each participant, as GFP peaks coincide with periods of maximal topographic stability and are therefore considered optimal sampling points for microstate extraction [43]. Scalp topographies at GFP peaks were extracted and submitted to a polarity-invariant modified k-means clustering algorithm, with EEG data normalized prior to clustering, and a minimum of 20 restarts performed to ensure convergence to the globally optimal solution [13]. Clustering was conducted across solutions ranging from 2 to 8 classes, from which the four-class solution was selected based on a mean global explained variance (GEV) of 84.7% [44]. Condition-level mean template maps were first computed separately for the EOR and ECR conditions, followed by group-level means for each diagnostic group (HC, MCI, AD), from which a single grand-mean template was derived by averaging across all group templates. Grand-mean maps were spatially sorted according to the canonical reference maps established by Custo et al. [16], and all group-level and individual maps were subsequently reordered in accordance with the grand mean to ensure topographically consistent labeling across participants and conditions [9]. The four microstate classes were labeled according to the canonical functional network attributions established through simultaneous EEG-fMRI studies [12,16], in which each microstate’s dynamics correlate with the BOLD signal of a specific resting-state network. These functional labels therefore denote the network whose activity co-varies with each microstate, rather than a focal cortical source underlying its scalp topography. Because the clustering was polarity-invariant, map polarity is arbitrary, and only the orientation of the topographic gradient is interpretable. The grand-mean template maps were then back-fitted to individual resting-state EEG recordings, exclusively at GFP peaks, to extract temporal microstate parameters for each participant [42].

**Figure 2:**
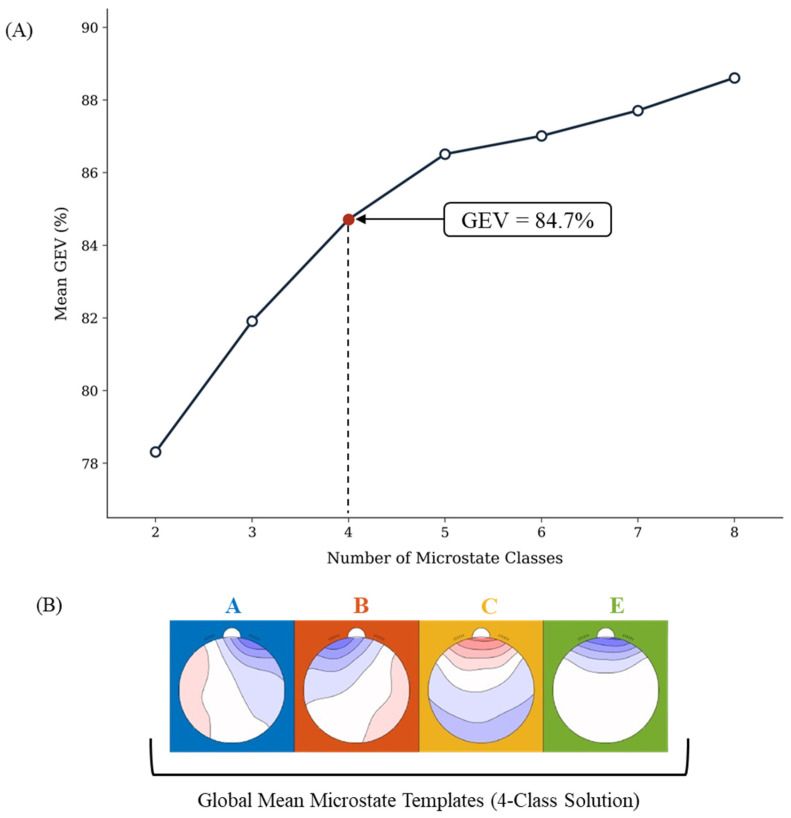
(**A**) Mean GEV as a function of the number of microstate classes (2–8). The four-class solution (GEV = 84.7%; red circle) was selected as optimal; the vertical dashed line marks the selected solution. (**B**) Four global mean microstate template maps derived from modified k-means clustering across all participants and back-fitted to individual rEEG recordings. Microstate classes are labeled A, B, C, and E following Koenig et al. [21]. Note: The functional network labels follow canonical EEG-fMRI attributions [12,16]; microstate topographies represent whole-scalp potential gradients and are not expected to spatially coincide with the cortical regions of the networks they index. Abbreviations: A, Auditory network microstate; B, Visual network microstate; C, Salience network microstate; E, Executive network microstate; GEV, global explained variance.

The four canonical grand-mean microstate template maps derived from the clustering procedure described above served as the basis for all microstate parameter computations (Figure 2). The mean GEV of 84.7% across all participants substantially exceeded the widely adopted adequacy criterion of 70%, confirming that the four-class solution sufficiently captured the dominant variance structure of the resting-state EEG signal. Although GEV increases monotonically with the number of clusters, the four-class solution was determined to offer an optimal balance between model fit and segmentation parsimony, consistent with its extensive application and validation in prior rEEG research [9,10,11,15,21,25,26]. Following back-fitting of the grand-mean template at GFP peaks, microstate class labels were assigned to each peak based on maximum spatial correlation with the template maps, with boundary midpoints between adjacent GFP peaks used to determine class assignment for intervening time points. Three canonical temporal parameters, duration, occurrence, and coverage, were subsequently extracted for each microstate class across both resting conditions [9,10,11,45].

### 2.6. Statistical Analysis for EEG Microstate

Topographic analyses were conducted in MATLAB (R2024a, accessed on 14 March 2026) using the MICROSTATELAB version 2.1 toolbox [42], while all temporal parameter analyses were performed in Python (Anaconda distribution (https://www.anaconda.com/download) (accessed on 28 March 2026)) within the JupyterLab environment, utilizing the SciPy, Pingouin, Pandas, and Scikit-posthocs libraries [45,46]. Statistical analyses proceeded in two complementary stages corresponding to the topographic and temporal dimensions of microstate characterization. Topographic differences in microstate maps were evaluated using topographic analysis of variance (TANOVA), a non-parametric randomization-based procedure implemented within MICROSTATELAB that assesses statistically significant differences in scalp field configuration without imposing distributional assumptions [15,42]. TANOVA was applied separately for the EOR and ECR resting conditions to evaluate overall between-group effects and pairwise topographic differences across HC, MCI, and AD for each microstate class independently. Within-group comparisons of microstate topography between EOR and ECR conditions were additionally conducted for each diagnostic group.

For temporal microstate parameters, duration, occurrence, and coverage, one-way ANOVAs were conducted separately for the EOR and ECR conditions, with diagnostic group (HC, MCI, AD) as the between-subjects factor and each microstate class analyzed independently. Significant omnibus effects were followed by post hoc pairwise comparisons with Bonferroni correction across three group pairs: HC vs. MCI, HC vs. AD, and MCI vs. AD. Non-parametric Kruskal–Wallis tests with Dunn post hoc corrections were additionally performed as sensitivity analyses to verify the robustness of parametric results. Effect sizes were reported as eta-squared (η^2^). Statistical significance was set at *p* < 0.05, with *p* < 0.01 considered highly significant.

## 3. Results

### 3.1. Microstate Cluster Evaluation

The GEV mean across all participants and resting conditions for the four-class grand mean template was 84.7% (SD = 10.1%). Under the EOR condition, mean GEV values were 83.4% (SD = 10.3%), 83.2% (SD = 9.5%), and 83.6% (SD = 9.4%) for HC, MCI, and AD, respectively. Under the ECR condition, corresponding values were 86.1% (SD = 10.8%), 85.1% (SD = 10.1%), and 87.0% (SD = 10.0%). One-way ANOVA confirmed no significant between-group difference in GEV under either the EOR (F (2177) = 0.023, *p* = 0.977) or ECR (F (2177) = 0.503, *p* = 0.606) conditions, indicating that microstate template fitting quality was fully comparable across diagnostic groups and did not introduce systematic bias into subsequent analyses.

### 3.2. Resting-State Microstate Topography

#### 3.2.1. Between-Group Topographic Differences (EOR)

**Figure 3:**
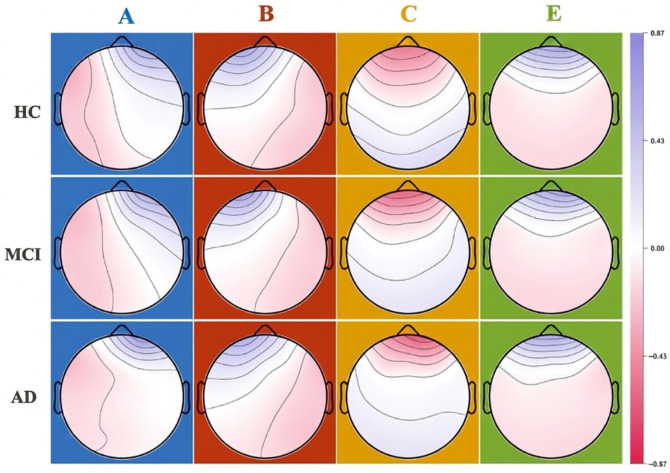
Grand-average rEEG microstate topographic maps under the EOR condition for HC, MCI, and AD groups. Columns represent microstate classes A (Auditory), B (Visual), C (Salience), and E (Executive); background colors are used consistently across all figures for class identification. Color bar indicates normalized scalp potential amplitude (−0.87 to 0.87). Between-group TANOVA results are reported in Table 1. Abbreviations: A, Auditory network microstate; AD, Alzheimer’s disease; B, Visual network microstate; C, Salience network microstate; E, Executive network microstate; HC, healthy controls; MCI, mild cognitive impairment.

Group-level topographic differences in resting-state microstate maps under the EOR condition were evaluated using TANOVA across HC, MCI, and AD, with each microstate class analyzed independently (Figure 3).

Under the EOR condition, four distinct microstate topographic patterns were identified across diagnostic groups (Figure 3), with the grand-average global mean templates (Figure 2) as the canonical spatial reference. Microstate A (Auditory) exhibited a right-anterior to left-posterior diagonal gradient, Microstate B (Visual) the mirror-image left-anterior to right-posterior diagonal, Microstate C (Salience) an anterior–posterior axial gradient, and Microstate E (Executive) a fronto-central, anterior-dominant configuration. These topographies reproduce the canonical microstate templates [12,16,21]; consistent with the polarity-invariant nature of microstate analysis, they are characterized by the orientation of their potential gradients rather than by the sign or focal location of scalp maxima. Progressive topographic variations across the HC–MCI–AD continuum were observed for Microstates A, C, and E, while Microstate B remained visually comparable across groups. TANOVA confirmed significant between-group topographic differences for Microstate A (*p* = 0.003), Microstate C (*p* = 0.004), and Microstate E (*p* = 0.036); Microstate B did not reach significance (*p* = 0.267; Table 1).

#### 3.2.2. Between-Group Topographic Differences (ECR)

**Figure 4:**
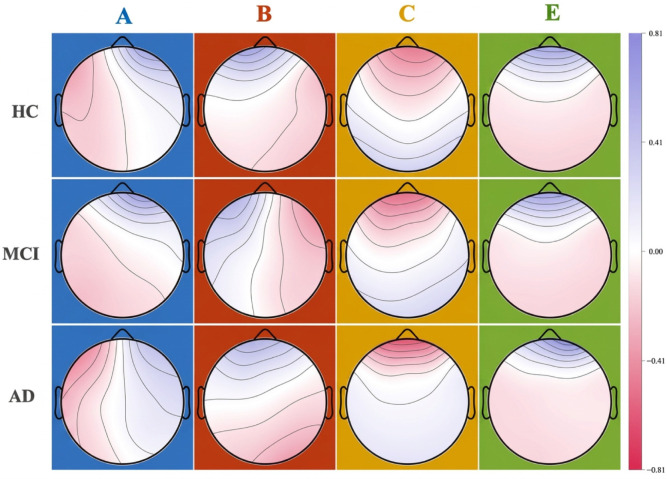
Grand-average rEEG microstate topographic maps under the ECR condition for HC, MCI, and AD groups. Columns represent microstate classes A (Auditory), B (Visual), C (Salience), and E (Executive); background colors are used consistently across all figures for class identification. Color bar indicates normalized scalp potential amplitude (−0.81 to 0.81). Between-group TANOVA results are reported in Table 1. Abbreviations: A, Auditory network microstate; AD, Alzheimer’s disease; B, Visual network microstate; C, Salience network microstate; E, Executive network microstate; HC, healthy controls; MCI, mild cognitive impairment.

Figure 4 displays the grand-average microstate topographic maps for each diagnostic group under the ECR condition, referenced to the global mean templates (Figure 2). Visually, all four microstate classes maintained topographic configurations consistent with their canonical patterns across HC, MCI, and AD, with no apparent group-level contour variations. In contrast to the EOR condition, TANOVA revealed no significant between-group topographic differences for any microstate class: Microstate A (Auditory) (*p* = 0.537), Microstate B (Visual) (*p* = 0.453), Microstate C (Salience) (*p* = 0.573), and Microstate E (Executive) (*p* = 0.453) all remained non-significant (Table 1). These results indicate that group-level topographic differences are not detectable under the ECR condition, suggesting that the ECR paradigm is less sensitive to disease-related topographic alterations across the HC–MCI–AD continuum.

#### 3.2.3. Within-Group Topographic Comparison (EOR vs. ECR)

**Figure 5:**
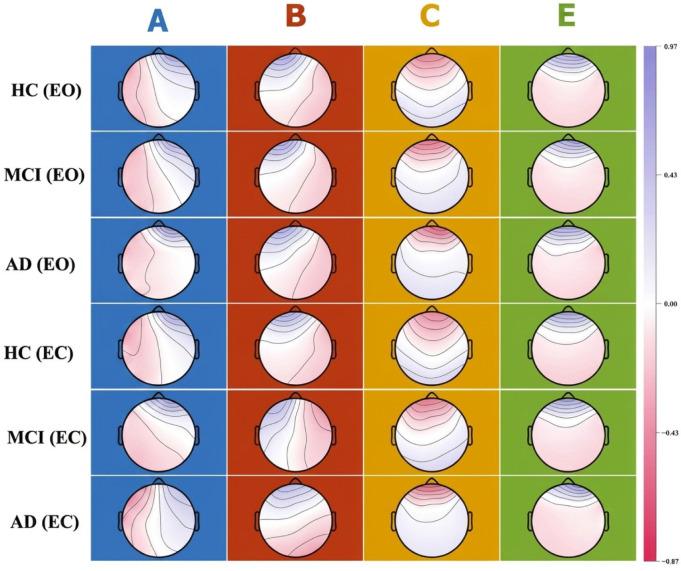
Grand-average rEEG microstate topographic maps for EOR and ECR conditions within each diagnostic group. Rows represent HC (EOR), MCI (EOR), AD (EOR), HC (ECR), MCI (ECR), and AD (ECR); columns represent microstate classes A (Auditory), B (Visual), C (Salience), and E (Executive). Background colors are used consistently across all figures for class identification. Color bar indicates normalized scalp potential amplitude (−0.87 to 0.87). Within-group TANOVA results are reported in Table 1. Abbreviations: A, Auditory network microstate; AD, Alzheimer’s disease; B, Visual network microstate; C, Salience network microstate; E, Executive network microstate; HC, healthy controls; MCI, mild cognitive impairment.

Within-group topographic comparisons between EOR and ECR conditions were conducted for each diagnostic group using TANOVA, with results summarized in Table 1 (Figure 5). In HC, a significant topographic difference between EOR and ECR was observed exclusively for Microstate B (Visual) (*p* = 0.042), while Microstates A (*p* = 0.507), C (*p* = 0.132), and E (*p* = 0.980) remained non-significant. A comparable pattern was identified in MCI, where only Microstate B demonstrated a significant EOR vs. ECR topographic difference (*p* = 0.038), with no significant effects observed for Microstates A (*p* = 0.505), C (*p* = 0.154), or E (*p* = 0.979). In contrast, the AD group exhibited a markedly broader pattern of condition-dependent topographic modulation, with significant EOR vs. ECR differences observed for Microstate A (*p* = 0.005), Microstate C (*p* = 0.043), and Microstate E (*p* = 0.007), while Microstate B did not reach significance (*p* = 0.118). These findings indicate that while HC and MCI show condition-dependent topographic modulation confined to the visual microstate, AD exhibits a broader EOR vs. ECR topographic divergence spanning auditory, salience, and executive network microstates.

### 3.3. Temporal Microstate Dynamics

**Figure 6:**
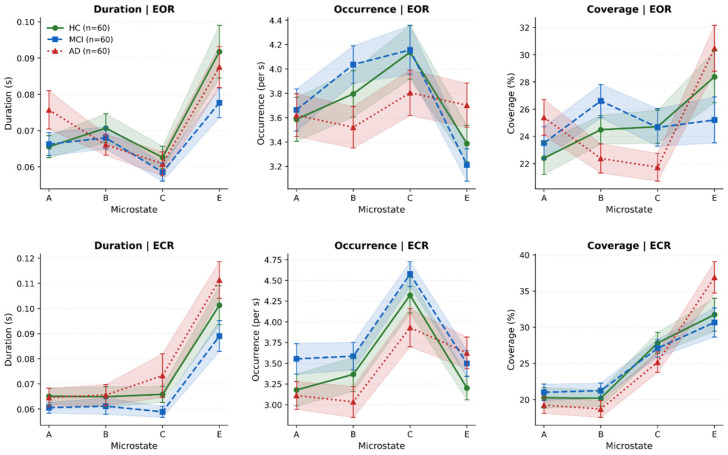
Group-level microstate temporal parameters, duration (s), occurrence (per s), and coverage (%), across HC, MCI, and AD under EOR (**upper panels**) and ECR (**lower panels**) conditions. Lines represent group means; shaded regions indicate ±1 standard error of the mean. A significant between-group difference was observed for Microstate B coverage under the EOR condition (F (2177) = 3.588, *p* = 0.030). Abbreviations: A, Auditory network microstate; AD, Alzheimer’s disease; B, Visual network microstate; C, Salience network microstate; E, Executive network microstate; ECR, eyes-closed resting; EOR, eyes-open resting; HC, healthy controls; MCI, mild cognitive impairment.

Group differences in microstate temporal parameters across HC, MCI, and AD are presented in Table 2, and the corresponding group-level distributions are illustrated in Figure 6. Under the EOR condition, one-way ANOVAs yielded no significant between-group differences in microstate duration or occurrence across any of the four classes. For microstate coverage, a significant group effect was observed exclusively for Microstate B (Visual) (F (2177) = 3.588, *p* = 0.030, η^2^ = 0.039). Post hoc pairwise comparisons with Bonferroni correction revealed that coverage of Microstate B was significantly greater in MCI than in AD (*p* = 0.031); no significant differences were observed between HC and MCI (*p* = 0.573) or HC and AD (*p* = 0.484). Notably, despite significant between-group topographic differences identified for Microstates A, C, and E under the EOR condition, no corresponding group differences were observed in their temporal parameters, suggesting a dissociation between the spatial and temporal dimensions of microstate alterations in the EOR state. Under the ECR condition, no significant between-group differences were identified for any temporal parameter across all four microstate classes, consistent with the absence of significant topographic group effects under this condition.

As illustrated in Figure 6, a consistent profile of temporal microstate dynamics was observed across all three diagnostic groups under both resting conditions. Across all groups, a characteristic pattern emerged: modest variation between Microstates A and B, a notable dip at Microstate C (Salience), and a sharp elevation at Microstate E (Executive), reflecting the longer mean duration of executive network microstate engagement. Under the EOR condition, AD showed a visually elevated duration at Microstate A relative to HC and MCI, while MCI displayed the lowest duration at Microstate E. Under the ECR condition, the rise in Microstate E duration was most pronounced in AD (111.32 ± 56.30 ms), followed by HC (101.31 ± 60.21 ms) and MCI (89.01 ± 47.31 ms). For occurrence, a prominent peak was consistently observed at Microstate C (Salience) across all groups and conditions, with MCI displaying the highest occurrence at Microstate C under both EOR (4.16 per s) and ECR (4.58 per s). For coverage, MCI exhibited the highest visual microstate (B) coverage under EOR, the only temporal parameter to reach statistical significance (F (2177) = 3.588, *p* = 0.030), while AD showed a progressive elevation in Microstate E coverage under ECR that approached but did not reach significance (*p* = 0.094). Despite these visual trends, most temporal parameters did not demonstrate statistically significant between-group differences, as detailed in Table 2.

## 4. Discussion

The present study investigated resting-state EEG microstate topography and temporal dynamics across the cognitive decline continuum, comprising HC, MCI, and AD, under EOR and ECR conditions in a balanced sample of 180 participants (n = 60 per group). To explore alterations in large-scale neural network organization across different stages of cognitive impairment, TANOVA was applied to assess between-group and within-group differences in microstate scalp topography, while one-way ANOVAs were used to examine group differences in microstate temporal parameters (duration, occurrence, and coverage). No significant between-group differences in mean GEV or microstate template fitting quality were observed under either resting condition (Table 2), indicating that the microstate differences identified between diagnostic groups are unlikely to reflect systematic variation in EEG data quality across groups. The following sections discuss the principal findings in the context of existing literature.

### 4.1. EOR-Specific Topographic Alterations in Cognitive Decline

The most notable finding of the present study is the selective sensitivity of the EOR condition to between-group differences in microstate topography across the HC–MCI–AD continuum. TANOVA revealed significant group effects for Microstate A (Auditory; *p* = 0.003), Microstate C (Salience; *p* = 0.004), and Microstate E (Executive; *p* = 0.036) under EOR, while no significant between-group topographic differences were identified under ECR across any microstate class (Table 1; Figure 3 and Figure 4). Barry et al. [47] established that EOR and ECR conditions yield fundamentally different EEG measures: the eyes-open state engages externally directed attentional and executive networks through heightened cortical arousal, while the eyes-closed state reflects alpha-dominant, internally directed processing. Consistent with this distinction, prior EEG microstate studies in the AD continuum have predominantly relied on ECR alone [24,48], and while some investigations recorded both conditions, only ECR portions were retained for analysis [49]. The present study directly addresses this gap, revealing that topographic group differences across the HC–MCI–AD continuum are selectively detectable under EOR and absent under ECR. The more integrated network organization characteristic of the EOR state [50] may amplify disease-related disruptions in large-scale network topography, which are attenuated by the more segregated, internally directed processing in the ECR condition. Furthermore, Zanesco et al. [20] demonstrated that both EOR and ECR reveal distinct microstate profiles even within normal-aging populations, underscoring the value of systematic, condition-specific analysis rather than reliance on a single paradigm.

The functional significance of these topographic group differences can be interpreted within the established framework linking EEG microstate classes to large-scale resting-state brain networks [9,12]. The significant EOR topographic group difference for Microstate A, associated with auditory network activity and characterized by a right-anterior to left-posterior diagonal gradient (Figure 3), is consistent with the known anatomical vulnerability of the medial temporal lobe in AD [51]. The hippocampus and entorhinal cortex, among the earliest structures affected by AD-related neurofibrillary pathology [52], are anatomically contiguous with the superior temporal gyrus and auditory cortices whose activity underlies the spatial profile of Microstate A [12]. Progressive medial temporal atrophy may therefore propagate topographic reorganization to adjacent auditory network microstates, becoming detectable at the scalp EEG level, particularly under the externally engaged EOR state.

The highly significant group effect for Microstate C (Salience; *p* = 0.004) aligns with converging evidence implicating salience network dysfunction as one of the earliest and most reproducible neurophysiological hallmarks of AD [24,26,27]. The salience network, anchored in the anterior cingulate cortex and bilateral insula, plays a fundamental role in coordinating attentional resource allocation and in regulating transitions between default mode and task-positive network states [12]. Disruption of salience microstate topography under EOR in MCI and AD may therefore reflect a core impairment in the neural mechanisms governing attentional filtering and network state regulation, consistent with the attentional and executive deficits characteristic of early cognitive decline [2,19,23,24]. Similarly, the significant EOR topographic group effect for Microstate E (Executive; *p* = 0.036) corresponds to established findings of frontoparietal executive network disruption in MCI and AD [23,24,26] and extends these observations to the resting-state EEG microstate domain under the EOR paradigm. By contrast, Microstate B (Visual) did not demonstrate significant between-group topographic differences under EOR (*p* = 0.267), suggesting that the spatial configuration of visual cortical network microstates, associated with negative BOLD activation in occipital visual areas [12], is relatively preserved across diagnostic groups at this stage of disease, consistent with the known relative sparing of occipital cortex in early-to-moderate AD [1,2].

### 4.2. Pathophysiological Correlates of Microstate Alterations in Cognitive Decline

The topographic microstate alterations observed across the HC–MCI–AD continuum are best understood as scalp-level correlates of the molecular and synaptic cascade that defines disease progression. The accumulation of amyloid-β plaques and neurofibrillary tau tangles follows a stereotyped spatial trajectory, advancing from medial temporal and limbic structures toward neocortical association areas [52], and preferentially burdens the highly connected cortical hub regions that anchor large-scale resting-state networks [53]. Because EEG microstates constitute the scalp-level signature of these same networks [12], the progressive disruption of network hubs provides a direct mechanistic link between AD neuropathology and the topographic microstate reorganization identified in the present study. At the synaptic level, this structural pathology is accompanied by degeneration of cholinergic projections from the basal forebrain and by glutamatergic excitotoxicity, both of which impair the rapid, coordinated switching between neural network states that microstate dynamics are thought to index [1,2]. Disruption of these neuromodulatory systems may explain why the AD group exhibited a broad loss of neural-state selectivity spanning auditory, salience, and executive microstates, whereas the MCI group, in which cholinergic tone and network integrity are comparatively preserved, displayed more circumscribed alterations. Within this framework, the salience and executive networks appear particularly vulnerable at early disease stages [24,26,27], consistent with the significant EOR topographic effects for Microstates C and E, before dysfunction generalizes to additional networks as neuropathology spreads. The EOR-specific topographic changes reported here therefore represent not isolated electrophysiological observations, but measurable scalp-level correlates of a progressive, network-specific pathophysiological process across the cognitive decline continuum.

### 4.3. Temporal Microstate Dynamics Across Cognitive Decline

Although significant EOR topographic group differences were identified for Microstates A, C, and E, no corresponding between-group differences were observed in the duration, occurrence, or coverage of any microstate class under either condition, with the sole exception of Microstate B coverage under EOR (Table 2; Figure 6). The finding that Microstate B coverage was significantly greater in MCI than in AD under EOR (MCI: 26.61 ± 9.47% vs. AD: 22.39 ± 8.28%; *p* = 0.031) is consistent with the compensatory neural hyperactivation hypothesis, wherein the early-impaired brain transiently over-recruits posterior visual network resources in response to emerging deficits in other resting-state networks [2,23,24,54]. Given that Microstate B has been associated with visual cortical processing and negative BOLD activation in occipital regions [12], the elevated visual microstate coverage in MCI may reflect increased reliance on visually anchored attentional processing as a compensatory strategy during early cognitive decline. This pattern mirrors the inverted-U model of neural compensation in neurodegeneration [55,56], wherein MCI may represent the peak of compensatory engagement before the progressive failure of this mechanism in AD.

The progressive reduction in visual microstate coverage from MCI to AD may further correspond to the occipital hypometabolism and posterior cortical involvement observed in moderate-to-advanced AD on fluorodeoxyglucose positron emission tomography (PET) imaging [9,57]. Importantly, the preserved topographic configuration of Microstate B across all three groups, evidenced by the non-significant TANOVA between-group effects, suggests that the spatial profile of visual network microstate activity remains relatively intact even as its temporal engagement is reduced, underscoring the complementary value of topographic and temporal microstate analyses in characterizing disease-related network changes [9,58].

The broad preservation of temporal microstate parameters across HC, MCI, and AD in the present study contrasts with some prior reports of significant alterations in microstate duration in AD [24,48,49]. This discrepancy may reflect differences in disease severity across study populations, as prior studies reporting significant changes in duration have often included more advanced AD cohorts [22,23,24]. It may also reflect the use of GFP-peak-restricted back-fitting without temporal smoothing in the present study, consistent with current best-practice recommendations [30], an approach that produces more conservative temporal parameter estimates than smoothing-based procedures applied in earlier investigations. Furthermore, the broad within-group variability observed across temporal parameters, particularly for Microstate E duration (HC SD = 56.24 ms; AD SD = 43.86 ms), may have reduced the statistical power to detect modest between-group differences. These findings collectively suggest that temporal microstate parameters may be less sensitive than topographic analysis to early-stage AD-related network alterations, a conclusion consistent with normative reference data demonstrating substantial between-study variability in temporal microstate parameters across resting conditions [58].

Two notable trends approaching statistical significance were observed under the ECR condition and are visually apparent in Figure 6. Microstate E duration was consistently elevated in AD relative to MCI (AD: 111.32 ± 56.30 ms vs. MCI: 89.01 ± 47.31 ms; *p* = 0.086; Table 2), suggesting prolonged executive network microstate engagement in AD during the internally directed resting state. Similarly, Microstate E coverage under ECR showed a visually prominent elevation in AD relative to HC and MCI (AD: 36.90 ± 16.73% vs. HC: 31.74 ± 17.64%; *p* = 0.094), possibly reflecting compensatory upregulation of frontoparietal resources in response to advancing network dysfunction [58]. Although neither trend survived the significance threshold in the present study, their consistent direction and proximity to significance suggest that executive network temporal microstate alterations under ECR may become detectable in larger cohorts and merit targeted investigation in future research.

### 4.4. Condition-Dependent Topographic Modulation: EOR vs. ECR

The within-group EOR vs. ECR topographic comparisons revealed a marked group-dependent pattern that further supports the neurophysiological relevance of the resting condition in EEG microstate research (Table 1; Figure 5). Krukow et al. [59] and Jao et al. [60] demonstrated that volitional eye opening, independent of visual stimulation, reorganizes large-scale brain network configurations, suggesting that the EOR state imposes active demands on neural state adaptation rather than reflecting passive sensory modulation. In HC and MCI, condition-dependent topographic modulation was confined to Microstate B (Visual; HC: *p* = 0.042; MCI: *p* = 0.038), reflecting the expected selective engagement of occipital visual networks during the open-eye state [12]. Mo et al. [61] further demonstrated that connectivity reorganization during eye-opening occurs more rapidly than during eye-closing, suggesting an asymmetric reorganization dynamic between the two resting states. The selective visual modulation in HC and MCI suggests that the cognitively intact and mildly impaired brain can efficiently and specifically adapt its microstate configurations in response to changing sensory states, a property consistent with the concept of neural state flexibility [9].

In marked contrast, the AD group demonstrated significant EOR vs. ECR topographic differences across Microstates A (*p* = 0.005), C (*p* = 0.043), and E (*p* = 0.007), with Microstate B remaining non-significant (*p* = 0.118). This expanded pattern of condition-dependent topographic modulation in AD may indicate a disease-related loss of neural-state selectivity, in which the AD brain fails to maintain the normal specificity of resting-state adaptation to changing sensory states. The involvement of auditory, salience, and executive network microstates in the EOR vs. ECR modulation profile in patients with AD may reflect progressive disruption of cholinergic and glutamatergic neuromodulatory systems, which are known to regulate large-scale network switching in AD [1,2]. Notably, these EOR vs. ECR differences are unlikely to reflect age-related artifacts, as Barry et al. [47] demonstrated that the fundamental neurophysiological distinctions between the two resting conditions remain stable across healthy aging. These findings suggest that the pattern of within-group EOR vs. ECR topographic modulation may carry additional diagnostic information beyond between-group comparisons alone, and warrants further investigation as a candidate marker of network dysregulation in AD.

The complete absence of between-group topographic differences under ECR, combined with the confined visual modulation in HC and MCI during the EOR vs. ECR comparison, suggests that the ECR condition imposes a degree of topographic uniformity across diagnostic groups that attenuates disease-related differences. This may be attributable to the alpha-oscillatory dominance of the ECR state, reflecting a shift toward internally directed default mode network processing [62,63], which partially normalizes resting-state microstate configurations across groups. These findings highlight the importance of systematically examining both EOR and ECR conditions in future EEG microstate studies, as reliance on ECR alone may substantially underestimate the sensitivity of microstate analysis to early network-level pathology in MCI and AD [27,49].

### 4.5. Strengths and Limitations of the Current Study

The present study offers several methodological strengths. The balanced three-group design with equal sample sizes (n = 60 per group; total N = 180) reduces the risk of group-size-related analytical bias. The systematic inclusion of both EOR and ECR conditions within a unified analytical framework is a notable methodological advance over prior studies limited to a single resting paradigm [24,49]. The application of MICROSTATELAB, a validated, open-source toolbox that implements current methodological recommendations, including GFP-peak-restricted back-fitting without temporal smoothing, ensures transparency, standardization, and reproducibility [30,42]. The combination of TANOVA for topographic analysis, one-way ANOVA with Bonferroni correction for temporal parameters, and non-parametric Kruskal–Wallis sensitivity analyses provides a comprehensive and rigorous statistical framework.

Several limitations should be acknowledged. The cross-sectional design precludes causal inference and longitudinal tracking of microstate changes. The 19-channel EEG system, while clinically practical, limits spatial resolution and precludes formal source localization, rendering microstate topographies reflections of mixed cortical source contributions that cannot be unambiguously assigned to specific neural generators. A related limitation concerns the recording reference. The linked-earlobe reference used here is not electrically neutral, as the earlobes themselves register volume-conducted brain activity, so the assumption of a zero-potential reference is not strictly met. This can reduce the spatial specificity of the topographies and may obscure whether left- or right-lateralized inferior temporal sources contribute to a given map, a consideration particularly relevant to the auditory microstate (Class A). Because all three groups were recorded and processed under an identical reference scheme, the between-group differences reported here are unlikely to reflect reference-related artifact, though the anatomical interpretation of individual topographies warrants caution. Reference-independent approaches, such as the Reference Electrode Standardization Technique (REST) or an average reference, would help resolve this ambiguity in future work.

Potential confounding variables, including medication effects, comorbid conditions, and sleep quality, were not systematically controlled and may have influenced resting-state microstate parameters. Single-center recruitment in a Korean clinical population may limit the generalizability of the findings to other ethnic and geographic contexts. The small effect size of the sole significant temporal finding (η^2^ = 0.039) underscores the need for replication in larger independent samples. Finally, the absence of microstate transition probability and syntax analysis in the present study represents a limitation relative to prior investigations, and future work incorporating such analyses could provide complementary insights into the sequential organization of network state transitions across the cognitive decline continuum.

## 5. Conclusions and Future Directions

Resting-state EEG microstate topographic analysis under the EOR condition demonstrated selective sensitivity to disease-related neural network alterations across the HC–MCI–AD continuum, identifying significant between-group topographic differences in auditory, salience, and executive network microstates that were absent under the ECR condition. A disease-specific broadening of condition-dependent topographic modulation was observed in AD, spanning microstates beyond the visual class that characteristically differentiated EOR from ECR in HC and MCI, suggesting a progressive loss of neural state flexibility with advancing cognitive decline.

Temporal microstate parameters were largely preserved across diagnostic groups, except for a selective reduction in visual network microstate coverage in AD relative to MCI under EOR, consistent with the failure of compensatory posterior network engagement in advanced disease. The spatiotemporal dissociation identified, in which topographic reorganization precedes detectable temporal parameter changes, suggests that microstate topographic analysis may offer earlier sensitivity to network-level pathology than temporal metrics alone. Collectively, these findings highlight the diagnostic potential of EOR-specific microstate topographic analysis as a non-invasive, methodologically rigorous tool for investigating large-scale network dysfunction in AD and underscore the importance of systematically examining resting conditions in future EEG microstate research.

Building on these findings, several directions merit future investigation. Longitudinal investigation of EOR microstate topographic trajectories is needed to determine whether the observed group differences constitute markers of progressive disease, and replication in larger, multi-center, and ethnically diverse cohorts is essential to establish generalizability. High-density EEG systems and source localization approaches would provide anatomical specificity to the reported topographic findings, while systematic investigation of associations between microstate topographic parameters and established cognitive biomarkers, including MMSE scores, cerebrospinal fluid amyloid and tau indices, and structural MRI atrophy measures, would clarify the clinical relevance of the identified alterations. Multimodal integration with functional MRI or PET could further elucidate the neurobiological substrates of the EOR-specific microstate reorganization observed in MCI and AD. Finally, incorporating microstate syntax and transition probability analyses into future studies would enable a more comprehensive characterization of resting-state network dynamics across the cognitive decline continuum.

## Figures and Tables

**Table 1:** Summary of TANOVA results for between-group and within-group comparisons across all microstate classes and resting conditions.

Microstate	Network	EOR *p*	ECR *p*	EOR vs. ECR HC	EOR vs. ECR MCI	EOR vs. ECR AD
A	Auditory	0.003 **	0.537	0.507	0.505	0.005 **
B	Visual	0.267 ns	0.453	0.042 *	0.038 *	0.118
C	Salience	0.004 **	0.573	0.132	0.154	0.043 *
E	Executive	0.036 *	0.453	0.980	0.979	0.007 **

Abbreviations: A, Auditory network microstate; AD, Alzheimer’s disease; B, Visual network microstate; C, Salience network microstate; E, Executive network microstate; ECR, eyes-closed resting; EOR, eyes-open resting; HC, healthy controls; MCI, mild cognitive impairment; TANOVA, topographic analysis of variance. * *p* < 0.05; ** *p* < 0.01; ns = not significant.

**Table 2:** Microstate temporal parameters across diagnostic groups under EOR and ECR conditions.

Parameter	Class	Cond.	HC Mean (SD)	MCI Mean (SD)	AD Mean (SD)	ANOVA	*p*	HC–MCI	MCI–AD	HC–AD
GEV (%)	—	EOR	83.37 (10.25)	83.18 (9.49)	83.57 (9.37)	F (2177) = 0.023	0.977	—	—	—
	—	ECR	86.08 (10.82)	85.09 (10.05)	86.97 (10.03)	F (2177) = 0.503	0.606	—	—	—
Duration (ms)	A	EOR	65.56 (23.54)	66.26 (24.52)	75.71 (40.68)	F (2177) = 2.058	0.131	—	—	—
		ECR	65.10 (24.65)	60.53 (16.91)	64.59 (28.63)	F (2176) = 0.660	0.518	—	—	—
	B	EOR	70.68 (30.62)	68.01 (23.96)	66.11 (22.18)	F (2176) = 0.470	0.626	—	—	—
		ECR	64.89 (31.02)	61.12 (24.82)	65.58 (31.67)	F (2174) = 0.397	0.673	—	—	—
	C	EOR	62.60 (23.88)	58.54 (18.96)	60.83 (25.55)	F (2177) = 0.471	0.625	—	—	—
		ECR	65.84 (24.88)	58.90 (17.24)	73.32 (66.63)	F (2176) = 1.749	0.177	—	—	—
	E	EOR	91.76 (56.24)	77.67 (31.66)	87.53 (43.86)	F (2177) = 1.547	0.216	—	—	—
		ECR	101.31 (60.21)	89.01 (47.31)	111.32 (56.30)	F (2177) = 2.487	0.086	—	—	—
Occurrence (/s)	A	EOR	3.59 (1.40)	3.66 (1.34)	3.62 (1.36)	F (2177) = 0.049	0.953	—	—	—
		ECR	3.18 (1.48)	3.55 (1.43)	3.11 (1.30)	F (2177) = 1.713	0.183	—	—	—
	B	EOR	3.80 (1.47)	4.04 (1.19)	3.52 (1.32)	F (2177) = 2.243	0.109	—	—	—
		ECR	3.37 (1.60)	3.59 (1.28)	3.03 (1.45)	F (2177) = 2.204	0.113	—	—	—
	C	EOR	4.14 (1.71)	4.16 (1.55)	3.80 (1.45)	F (2177) = 0.951	0.388	—	—	—
		ECR	4.32 (1.66)	4.58 (1.15)	3.93 (1.78)	F (2177) = 2.635	0.075	—	—	—
	E	EOR	3.39 (1.16)	3.21 (1.02)	3.70 (1.41)	F (2177) = 2.554	0.081	—	—	—
		ECR	3.20 (1.10)	3.50 (1.20)	3.63 (1.47)	F (2177) = 1.757	0.176	—	—	—
Coverage (%)	A	EOR	22.41 (9.25)	23.52 (9.27)	25.40 (10.12)	F (2177) = 1.501	0.226	—	—	—
		ECR	20.25 (10.97)	21.01 (8.82)	19.24 (9.02)	F (2177) = 0.509	0.602	—	—	—
	B	EOR	24.49 (8.11)	26.61 (9.47)	22.39 (8.28)	F (2177) = 3.588	0.030	0.573	0.031	0.484
		ECR	20.19 (8.66)	21.23 (8.07)	18.69 (8.98)	F (2177) = 1.324	0.269	—	—	—
	C	EOR	24.72 (9.49)	24.66 (10.71)	21.74 (7.88)	F (2177) = 1.955	0.145	—	—	—
		ECR	27.83 (11.25)	27.10 (9.47)	25.17 (10.88)	F (2177) = 1.010	0.366	—	—	—
	E	EOR	28.38 (14.71)	25.21 (13.06)	30.48 (13.07)	F (2177) = 2.269	0.106	—	—	—
		ECR	31.74 (17.64)	30.67 (15.58)	36.90 (16.73)	F (2177) = 2.396	0.094	—	—	

Note: Values are Mean (Standard Deviation). The *p*-values indicate statistical significance (*p* < 0.05). Post hoc *p*-values are Bonferroni-corrected for three group pairs. — = not applicable. Abbreviations: A, Auditory network microstate; AD, Alzheimer’s disease; ANOVA, analysis of variance; B, Visual network microstate; C, Salience network microstate; Cond., condition; E, Executive network microstate; ECR, eyes-closed resting; EOR, eyes-open resting; GEV, global explained variance; HC, healthy controls; MCI, mild cognitive impairment; SD, standard deviation.

## Data Availability

The data supporting the findings of this study are available upon reasonable request from the corresponding author, Y.C.Y., via email. Due to confidentiality agreements and institutional policies protecting sensitive patient information from the hospital where the study was conducted, the data cannot be made publicly available. Requests will be evaluated to ensure compliance with ethical and legal standards.

## Supplementary Materials

The following supporting information can be downloaded at: https://www.mdpi.com/article/10.3390/jcm15155863/s1, Figure S1: 19 Scalp Electrodes Positioned based on the International 10–20 System.
