# Supplementary material for: EEG Microstate Alterations in Eyes-Open and Eyes-Closed Resting States Across the Alzheimer’s Disease Continuum
Source: J Clin Med. 2026 Jul 27;15(15):5863. doi: 10.3390/jcm15155863 (PMC13467254; doi:10.3390/jcm15155863)

Supplementary Figure S1 19 Scalp Electrodes Positioned based on the International 10-20 System

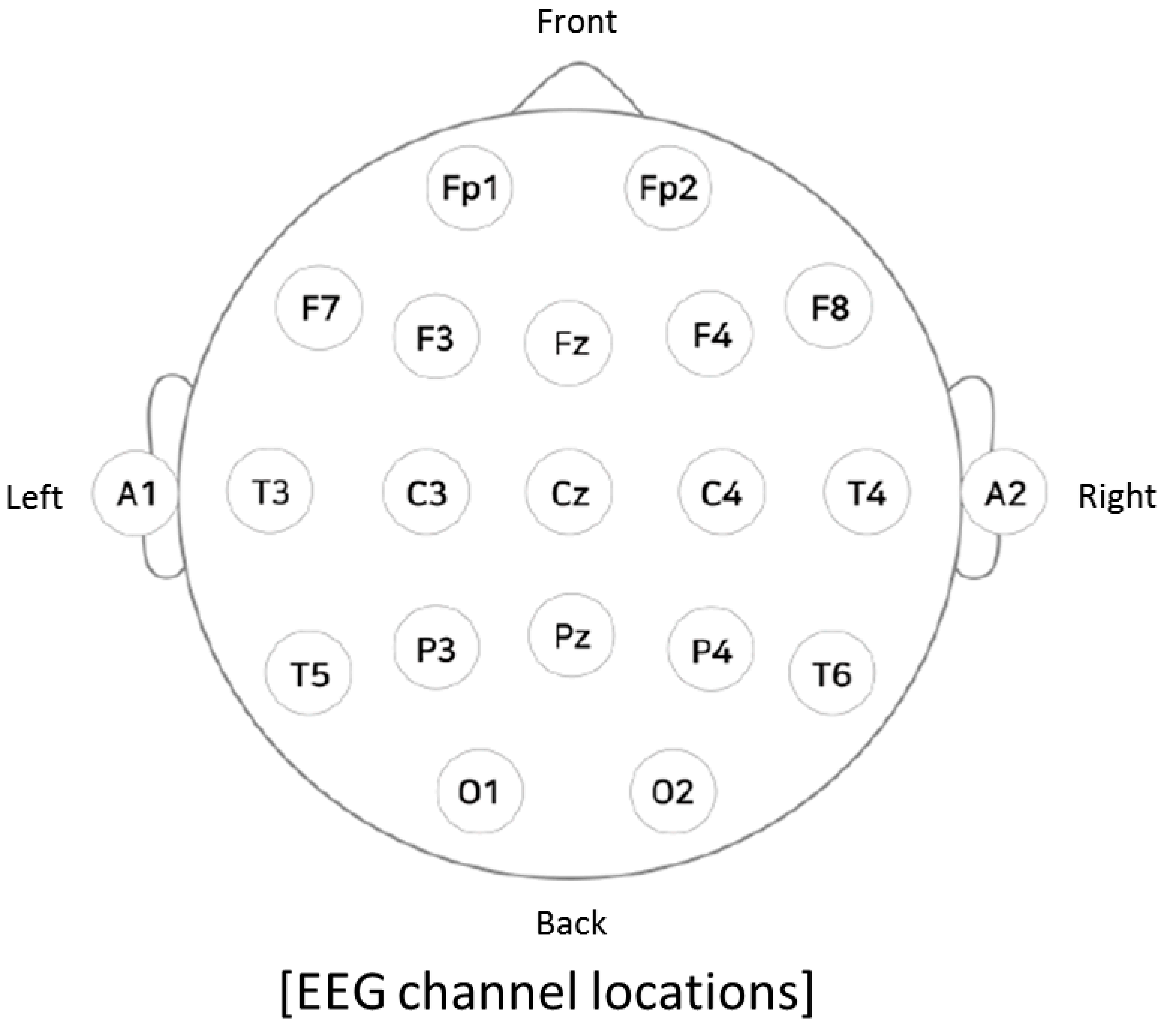

Supplement: Supplementary file 1 [file jcm-15-05863-s001.zip › jcm-4463588-supplementary.pdf]
